# Emerging Role of Proteases in the Pathogenesis of Chronic Rhinosinusitis with Nasal Polyps

**DOI:** 10.3389/fcimb.2017.00538

**Published:** 2018-01-12

**Authors:** Dawei Wu, Yongxiang Wei, Benjamin S. Bleier

**Affiliations:** ^1^The Department of Otolaryngology, Massachusetts Eye and Ear Infirmary, Harvard Medical School, Boston, MA, United States; ^2^The Department of Otorhinolaryngology, Beijing Anzhen Hospital, Capital Medical University, Beijing, China

**Keywords:** chronic rhinosinusitis, nasal polyps, eosinophil, protease, epithelium

## Abstract

Chronic rhinosinusitis with nasal polyps (CRSwNP) is a heterogeneous upper airway disease with multiple etiologies. Clinically, CRSwNP can be classified into either eosinophilic or non-eosinophilic subtypes. The eosinophilic phenotype of CRSwNP is widely thought to be highly associated with recurrence of nasal polyps or surgical failure. Epithelial cells have a crucial role in the development of Th2-biased airway diseases. Recent studies have shown that a wide range of external stimuli such as allergens and microorganisms can elicit the release of epithelial-derived Th2-driving cytokines and chemokines. Protease activity is a feature common to these multiple environmental insults and there is growing evidence for the concept that an imbalance of proteases and protease inhibitors in the epithelial barrier leads to both the initiation and maintenance of chronic eosinophilic airway inflammation. In this review, we analyze recent work on the role of proteases in the development of the sinonasal mucosal type 2 immune response with an emphasis on the molecular pathways promoting adaptive Th2 cell immunity.

## Introduction

Chronic rhinosinusitis is a chronic inflammatory upper airway disease characterized by 12 weeks of typical symptoms including nasal discharge, congestion, facial pressure or pain, and olfactory disorder (Fokkens et al., 2012). Chronic rhinosinusitis with nasal polyps (CRSwNP), a multifactorial and highly heterogeneous upper airway disease, is a severe phenotype of chronic rhinosinusitis and presents with distinct immunological and histopathological features compared with chronic rhinosinusitis without nasal polyps (CRSsNP).

Despite aggressive medical therapy or radical endoscopic sinus surgical treatment, many patients with CRSwNP tend to be poorly controlled and have a high recurrence rate (Wynn and Har-El, 2004; Mendelsohn et al., 2011; Baguley et al., 2014; DeConde et al., 2017). Several factors which associate with a worse outcome or recurrence risk have been identified, such as high tissue eosinophil infiltration, more severe preoperative disease (i.e., a higher CT score), and a series of comorbid disease (i.e., aspirin-exacerbated respiratory disease (AERD), allergic asthma and cystic fibrosis) (Desrosiers, 2004; Tosun et al., 2010; Mortuaire et al., 2015; Ta and White, 2015; Tipirneni and Woodworth, 2017; Wu et al., 2017).

Clinically, CRSwNP is classified into two phenotypes based on the dominant inflammatory cell type in tissues: eosinophilic CRSwNP (ECRSwNP) and non-eosinophilic CRSwNP (NECRSwNP) (Cao et al., 2009; Shah et al., 2016; Wu et al., 2016; Cho S.-W. et al., 2017). In western countries, the majority of patients with CRSwNP (80–88%) have prominent tissue eosinophilia, edema formation, and a type 2 helper T-cell (Th2) dominant immune response (Bateman et al., 2005; Fokkens et al., 2005; Van Zele et al., 2006). CRSwNP may be associated with asthma and aspirin intolerance (Fokkens et al., 2012; Stevens et al., 2017). However, at least half of patients with CRSwNP in East Asian countries including China, Korea and Japan have a non-eosinophilic phenotyps of nasal polyps characterized by Th1/Th17-dominant inflammation (Kim et al., 2007; Zhang et al., 2008; Cao et al., 2009; Ikeda et al., 2013).

The past decade has witnessed a change in the understanding of mechanisms underlying eosinophilic airway diseases from a paradigm in which allergen-independent, e.g., Th2 cells are the primary drivers, to one in which production of epithelial-derived chemokines and cytokines by dysfunctional respiratory epithelium are the primary orchestrators of the eosinophilic immune response (Hammad and Lambrecht, 2015; Pfeffer and Corrigan, 2017). A large range of both endogenous and extrinsic stimuli can activate the epithelial cell and elicit the release of epithelial-derived chemokines and cytokines which, in turn, induce the type 2 immune response (Hammad and Lambrecht, 2015; Schleimer and Berdnikovs, 2017). External stimuli, including allergen, fungus, *Staphylococcus aureus* and microbiome disturbance have been posited as significant contributing factors in CRSwNP pathophysiology and have been implicated in driving Th2-biased airway disease (Sachse et al., 2010; Clark et al., 2013; Madeo and Frieri, 2013; Ou et al., 2014; Lan et al., 2016; Orlandi et al., 2016; Tomassen et al., 2016; Schleimer, 2017).

Protease activity is a common unifying feature of many of these environmental insults suggesting an underlying common etiopathogenesis (Sokol et al., 2008; Gregory and Lloyd, 2011; Stentzel et al., 2017; Teufelberger et al., 2017). Airborn allergens, such mites, pollen, as well as microorganisms, such as bacteria, rhinovirus, and influenza virus, and fungi are major sources of exogenous proteases (Reed and Kita, 2004; Sokol et al., 2008; Costenaro et al., 2011; Takai and Ikeda, 2011; Kesic et al., 2012). The innate immune response to these exogenous proteases seems to play a crucial role during the development of Th2-biased immune response (Kamijo et al., 2013; Hara et al., 2014; Snelgrove et al., 2014; Teufelberger et al., 2017). It therefore follows that an imbalance of proteases and protease inhibitors in the epithelial barrier may lead to the initiation and maintainancc of eosinophilic inflammation in CRSwNP and therefore be a central driver of eosinophilic airway disease (Kouzaki et al., 2017; Pfeffer and Corrigan, 2017).

This review will summarize the current knowledge on the role of proteases during the development of the sinonasal mucosal type 2 immune response, with an emphasis on the molecular pathways initiating the innate type 2 cell response and then promoting adaptive Th2 cell immunity. This is followed by a discussion of the dysfunctional regulation of proteases and proteases inhibitors in the epithelial barrier.

### Mechanisms of the activation of the airway epithelial cells upon external protease exposure

**Figure 1:**
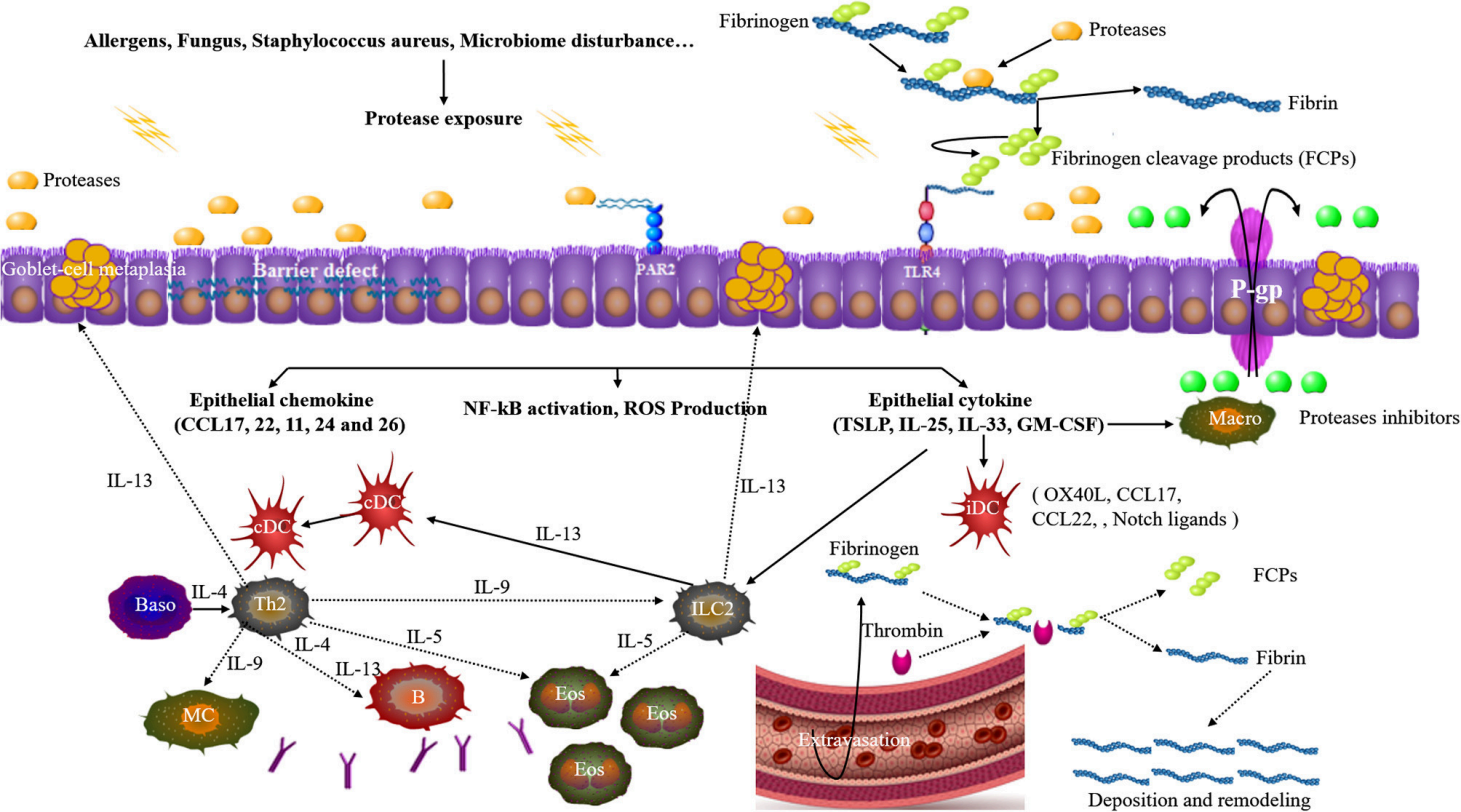
Upon allergen proteases exposure, junctional proteins among epithelial cells are disrupted. Allergen proteases can directly react with protease-activated receptor 2 (PAR2). Allergen proteases cleave the serum factor fibrinogen, thus releasing fibrinogen cleavage products (FCPs) which can activate toll-like receptor 4 (TLR4). Epithelial cells get activated to produce and release pro-Th2 cell chemokines and cytokines which instruct immature dendritic cells (iDC) and activate ILC2s. Additionally, the activation of these receptors will also induce NF-kB activation, ROS production. Th2 cells and ILC2s are activated and promote the eosinophilia, production of IgE and goblet-cell metaplasia. Allergen exposure is generally accompanied by fluid extravasation and thrombin also generates FCPs from fibrinogen, thus triggering TLR4. P-glycoproteins (P-gp) in the epithelial cells promote the efflux of protease inhibitors to suppress the allergen proteases. *cDC* classical DC, *Macro* macrophage, *Baso* basophils, *MC* mast cell.

Cysteine and or serine proteases occur in some groups of airborne mite, pollen, cockroach, fungi, and *Staphylococcus aureus* (Asokananthan et al., 2002; Reed and Kita, 2004; Jacquet, 2011; Takai and Ikeda, 2011; Balenga et al., 2015; Kale et al., 2017; Stentzel et al., 2017; Teufelberger et al., 2017). Allergen derived proteases interact with epithelial cells through three principle pathways: direct effects on junctional proteins, reacting with cell surface protease-activated receptors (PARs), and toll-like receptor 4 (TLR4)-dependent epithelial activation. An integrated mechanism is summarized and illustrated in Figure 1.

Allergen source-derived proteases (both cysteine and serine protease) can directly degrade tight junctions in the epithelium (Wan et al., 1999, 2001; Tai et al., 2006; Runswick et al., 2007; Hirasawa, 2010; Kale et al., 2017) and increase the accessibility of microorganisms and antigens to the underlying lamina propria and connective tissue thereby triggering strong innate immune responses to allergens (Gregory and Lloyd, 2011). It has been reported that the levels of occludin, E-cadherin, and zonula occludens-1 (ZO-1) were all reduced in mature polyps derived from patients with CRSwNP. Moreover, aquaporin 5, a marker of epithelial differentiation, was obviously reduced in sinonasal samples of patients with CRSwNP when compared with levels in CRSsNP or control subjects (Shikani et al., 2014).

Apart from direct effects on junctional epithelial proteins, environmental proteases can interact with PARs in the airway to stimulate the proliferation and migration of innate and adaptive leukocytes (Reed and Kita, 2004). PARs are a novel family of seven-transmembrane G protein-coupled receptors that are widely expressed in human airway epithelium. There are four types of PARs (PAR1, PAR2, PAR3, and PAR4) which play an integral role in defending against environmental proteases (Coughlin and Camerer, 2003; Reed and Kita, 2004). Several reports have linked PAR activation to the allergic immune response (Kheradmand et al., 2002; Jacquet, 2011). Exogenous proteases from house dust mite (HDM), cockroach or *Alternaria alternate* were shown to play an important role in allergy development, partly by activating PAR-2 signaling in the epithelial cells (de Boer et al., 2014). In CRSwNP, airborne fungal proteases can activate both PAR-2 and PAR-3 leading to the proliferation and migration of inflammatory cells (Shin et al., 2006). Furthermore, the level of the PAR-2 in cultured primary nasal epithelial cells and nasal polyps from patients with ECRSwNP was significantly increased as compared with NECRSwNP and controls (Kouzaki et al., 2016). However, in patients with allergic fungal rhinosinusitis, only PAR-3 showed statistically significant differential expression compared to non-diseased controls (Ebert et al., 2014).

*Staphylococcus aureus* is a versatile bacteria frequently found colonizing patients with Th2-biased diseases such CRSwNP and asthma (Bachert et al., 2010; Sachse et al., 2010). Several endotypes of chronic rhinosinusitis have been identified based on the presence of S. aureus enterotoxin(SE)- specific IgE (Bachert and Akdis, 2016; Tomassen et al., 2016). The presence of SE-specific IgE associates with intense eosinophilic inflammation in CRSwNP, high IgE concentration and comorbid asthma (Bachert et al., 2010; Tomassen et al., 2016). Recently, serine protease like protein D (SplD) and other closely related proteases secreted by S. aureus have been identified as inducers of allergic asthma in both humans and mice (Stentzel et al., 2017). Furthermore, SplD-induced Th2-biased inflammatory response and IgE production in the airway inflammation were largely dependent on the IL-33/ST2 axis and independent of TLR4 and PAR-2 signaling (Teufelberger et al., 2017).

TLR activation has been the subject of intense study with respect to its role in protease mediated airway inflammation. The coagulation system has been implicated in eosinophilic airway diseases, such as asthma and CRSwNP as a result of collagen deposition and airway remodeling, (Shimizu et al., 2011; Lambrecht and Hammad, 2013; Kim et al., 2015). Millien et al. found that activation of the coagulation cascade by allergen-derived proteases is an important factor promoting asthma-like changes in mice. Allergen proteases can cleave the serum factor fibrinogen, thus releasing FCPs which directly activate TLR4 signaling (Millien et al., 2013). The development of an asthma-like condition caused by house-dust mites challenge relies on the expression of TLR4 on lower airway epithelial cells (Hammad et al., 2009). Furthermore, thrombin, the classic activator of coagulation, can also cleave fibrinogen into FCPs resulting in further upregulation of the TLR4 pathway. A recent study identifies a programmed cell death 1 ligand 2^+^ (PD-L2^+^) DC phenotype which accounts for the induction of Th2 cell response upon protease allergens exposure and fibrinogen-cleavage products can promote the generation of PD-L2^+^ DC through TLR4 (Cho M. et al., 2017). These studies suggest that TLR4 plays a critical role in the allergic response upon exposure to exogenous proteases.

A study by Seung-Heon Shin et al. showed that airborne fungi induced the activation of nasal polyp epithelial cell and TLR expression (TLR2, TLR3 and TLR4). Cytokine production was, in turn, suppressed by protease inhibitors and anti-TLR4 antibodies (Shin and Lee, 2010).

### Imbalance and dysfunctional regulation of proteases and proteases inhibitors in the epithelial barrier of CRSwNP

Recently, a study showed that an imbalance of proteases and protease inhibitors within the epithelial barrier contributes to the pathogenesis of eosinophilic chronic rhinosinusitis (Kouzaki et al., 2017). Barrier defects might be induced by damage to key proteins that comprise tight or adherent junctions secondary to increased or unopposed protease activity. These findings suggest that individual susceptibility to protease mediated inflammation may arise from the inability to adequately mitigate exogenous protease mediated epithelial damage. A recent review (Schleimer and Berdnikovs, 2017) suggested that cystatin A and SPINK5 (a cysteine and serine protease inhibitor, respectively) possess important roles in protecting the airway epithelium against environmental proteases. Furthermore, SPINK5 can protect PARs which are expressed on multiple cell types in the nasal epithelium from environmental proteases (Hershenson, 2007). Furthermore, SPINK5 is thought to regulate the function of numerous proteases that might compromise the barrier (Tieu et al., 2009). What's more, both human and animal studies have showed that SPINK5 mutations are associated with chronic inflammation in epithelium (Cookson, 2004; Moffatt, 2004).

P-glycoprotein (P-gp) has been reported as a key immunoregulator of eosinophilic inflammation in both CRSwNP and CRSsNP (Bleier et al., 2013; Feldman et al., 2013; Cheng and Bleier, 2016). Protease inhibitors have been reported to induce the expression of P-gp suggesting that an imbalance in the protease system may further exacerbate inflammation through the induction of P-gp expression(Perloff et al., 2000; Huang et al., 2001; Chandler et al., 2003; Zastre et al., 2009). Additionally, some protease inhibitors have been shown to function as P-gp substrates further strengthening the link between protease inhibitors and P-gp (Chaillou et al., 2002; Meaden et al., 2002) (Zhang and Benet, 1998). While disequilibrium of both P-gp expression and proteases inhibitors within the nasal mucosa may play an interrelated role in CRSwNP, further studies are needed to explore this possible function.

### Summary and perspectives

In patients with CRSwNP, exogenous allergen and microorganism derived proteases play a crucial role in the development of type 2 immune response at the mucosal surface. Through direct effects on junctional proteins, binding to cell surface PARs, TLR4-dependent epithelial activation, disruption of barrier function, and P-gp activation, proteases both initiate and maintain the inflammation characteristic of Th2 mucosal disease. It has been proposed that drugs targeting protease function (Verma et al., 2016) in nasal mucus to restore the balance between proteases and protease inhibitors (Pfeffer and Corrigan, 2017) may represent an important potential therapeutic strategy in patients with CRSwNP and other eosinophilic airway diseases. However, more studies are required to explore the exact role of the protease and protease inhibitor axis in CRSwNP.

## Author contributions

DW drafted the manuscript. Both YW and BB reviewed the manuscript and provided revisions.

### Conflict of interest statement

The senior author has a patent related to P-gp modulation in CRSwNP. The other authors declare that the research was conducted in the absence of any commercial or financial relationships that could be construed as a potential conflict of interest.

## Abbreviations

CRSwNP: Chronic rhinosinusitis with nasal polyps
CRSsNP: chronic rhinosinusitis without nasal polyps
ECRSwNP: eosinophilic chronic rhinosinusitis with nasal polyps
NECRSwNP: non-eosinophilic chronic rhinosinusitis with nasal polyps
Th2: type 2 helper T-cell
PARs: protease-activated receptors
TLR4: toll-like receptor 4
ZO-1: Zonula occludens-1
HDM: house dust mite
SpID: serine protease like protein D
FCPs: fibrinogen cleavage products
P-gp: P-glycoprotein.
